# Antibiofilm Activity of a Broad-Range Recombinant Endolysin LysECD7: In Vitro and In Vivo Study

**DOI:** 10.3390/v12050545

**Published:** 2020-05-15

**Authors:** Mikhail V. Fursov, Radmila O. Abdrakhmanova, Nataliia P. Antonova, Daria V. Vasina, Anastasia D. Kolchanova, Olga A. Bashkina, Oleg V. Rubalsky, Marina A. Samotrueva, Vasiliy D. Potapov, Valentine V. Makarov, Sergey M. Yudin, Alexander L. Gintsburg, Artem P. Tkachuk, Vladimir A. Gushchin, Evgenii O. Rubalskii

**Affiliations:** 1 Aerobiological Laboratory, Antimicrobial Agents Laboratory, State Research Center for Applied Microbiology and Biotechnology, 142279 Obolensk, Russia; mikhail.fursov88@gmail.com (M.V.F.); kolchanova_a@list.ru (A.D.K.); potapovvd@mail.ru (V.D.P.); 2 Astrakhan State Medical University, 414000 Astrakhan, Russia; radmilaazo@mail.ru (R.O.A.); bashkina1@mail.ru (O.A.B.); rubalsky.innovation@gmail.com (O.V.R.); ms1506@mail.ru (M.A.S.); 3 N.F. Gamaleya National Research Centre for Epidemiology and Microbiology, Ministry of Health of the Russian Federation, 123098 Moscow, Russia; northernnatalia@gmail.com (N.P.A.); d.v.vasina@gmail.com (D.V.V.); gintsburg@gamaleya.org (A.L.G.); artem.p.tkachuk@gmail.com (A.P.T.); 4 Lomonosov Moscow State University, 119991 Moscow, Russia; 5 A.N. Bach Institute of Biochemistry, Research Center of Biotechnology of the Russian Academy of Sciences. 33, bld. 2 Leninsky Ave., 119071 Moscow, Russia; 6 Center for Strategic Planning of the Ministry of Health of the Russian Federation, 119435 Moscow, Russia; makarovvalentine@gmail.com (V.V.M.); info@cspmz.ru (S.M.Y.); 7 Infectiology Department, I. M. Sechenov First Moscow State Medical University, 119146 Moscow, Russia; 8 G.N. Gabrichevsky Moscow Research Institute of Epidemiology and Microbiology, 125212 Moscow, Russia; 9 Department of Cardiothoracic, Transplantation and Vascular Surgery, Hannover Medical School, 30625 Hannover, Germany; 10 Lower Saxony Centre for Biomedical Engineering, Implant Research and Development, 30625 Hannover, Germany

**Keywords:** endolysin, biofilm degradation, drug-resistant bacteria, implant-associated infection model, animal trial

## Abstract

Surfaces of implanted medical devices are highly susceptible to biofilm formation. Bacteria in biofilms are embedded in a self-produced extracellular matrix that inhibits the penetration of antibiotics and significantly contributes to the mechanical stability of the colonizing community which leads to an increase in morbidity and mortality rate in clinical settings. Therefore, new antibiofilm approaches and substances are urgently needed. In this paper, we test the efficacy of a broad-range recombinant endolysin of the coliphage LysECD7 against forming and mature biofilms. We used a strong biofilm producer—*Klebsiella pneumoniae* Ts 141-14 clinical isolate. In vitro investigation of the antibacterial activity was performed using the standard biofilm assay in microtiter plates. We optimized the implantable diffusion chamber approach in order to reach strong biofilm formation in vivo avoiding severe consequences of the pathogen for the animals and to obtain a well-reproducible model of implant-associated infection. Endolysin LysECD7 significantly reduced the biofilm formation and was capable of degrading the preformed biofilm in vitro. The animal trials on the preformed biofilms confirmed these results. Overall, our results show that LysECD7 is a promising substance against clinically relevant biofilms.

## 1. Introduction

New antibacterial strategies and agents are urgently needed due to the emergence and development of resistance to conventional antibiotics. Formation of bacterial biofilm (BF) is a risk factor of infectious disease development. The antibiotic response patterns in BF significantly vary from those specific for the planktonic cells [1]. The bacterial community became more tolerant to antibacterial substances and more mechanically stable [2]. Thus, clinical treatments with antibiotics concentrations determined for planktonic bacteria may result in persistent infection formation. Biofilms are prevalent in 78.2% chronic wounds [3]. Severe recalcitrant infections are usually expected as a consequence of biofilm formation in patients with medical implants. A positive outcome for the majority of patients with formed biofilms on implanted medical devices is only possible after surgical replacement of the implant [4,5]. However, such therapy itself carries a risk for patients’ life. Today, more than 80% of microbial infections are related to BF formation [6], thus new antibiofilm drugs are needed.

Bacteriophages can be used as a promising source of substances active against pathogenic and opportunistic bacteria. A number of phage-derived enzymes with antibacterial properties were already isolated and tested both in vitro and in vivo [7,8,9,10,11,12,13,14,15,16,17]. Phage endolysins and depolymerases are the main candidates for a search of the suitable hits [18]. Previously, we have synthesized and successfully tested in vitro few recombinant endolysins [19]. One of them, LysECD7, showed outstanding broad range activity against the planktonic forms and was proposed for further investigation of its broad antibacterial efficacy [20].

The aim of this study was to determine whether LysECD7 endolysin could be used for the therapy of bacterial biofilms in vitro and in vivo. The antibacterial activity against preformed biofilms of clinically isolated multidrug-resistant strain of *Klebsiella pneumoniae* was tested. LysECD7 acted against emerging and formed biofilms both in vitro and in vivo. We consider LysECD7 to be a promising agent with strong antibiofilm activity suitable for local or systemic application.

## 2. Materials and Methods

### 2.1. Bacterial Strain

Multidrug-resistant *Klebsiella pneumoniae* strain Ts 141-14 was initially isolated from the urine of a patient, hospitalized in the Medical and Rehabilitation Center, Moscow, in 2014. The strain is stored in the collection of the N.F. Gamaleya Federal Research Center for Epidemiology and Microbiology, Ministry of Health of the Russian Federation in 30% glycerol solution frozen stocks of bacterial cells at −80 °C. Bacteria were grown on nutrient media GRM 1 (SRCAMB, Obolensk, Russia) and Mueller–Hinton broth and agar (Thermo Fisher Scientific, Waltham, MA, USA) at 37 °C under aerobic conditions. GRM 1 media (Fish powder hydrolysate broth) contains pancreatic hydrolyzate of fish meal (15 g/L), pancreatic hydrolyzate of casein (10 g/L), yeast extract (2 g/L), sodium chloride (3.5 g/L), glucose (1 g/L).

### 2.2. Construct Cloning

LysECD7 protein (NCBI AN: YP_009602067.1) was obtained as described before [20], synthetic gene was used for investigations. Briefly, LysECD7′s initial coding sequence was artificially synthesized in a pAL-TA commercial vector (Evrogen Ltd., Moscow, Russia). Thereafter endolysin ORF was amplified from a pALTA-LysECD7 clone and integrated into the expression vector pET-42b(+) (Evrogen Ltd., Moscow, Russia), resulting in a pET42b-LysECD7-8his plasmid. All constructs were checked for errors via Sanger sequencing.

### 2.3. Recombinant Expression and Purification of LysECD7-8his

Expressed endolysin contained a C-terminal 8-His tag for affinity purification. The expression vectors were introduced into the competent *E. coli* cells, strain BL21(DE3) pLysS (chloramphenicol resistance), using a heat shock transformation protocol. The *E. coli* cells were grown in an LB broth (37 °C, 240 rpm) to an OD600 value of 0.55–0.65 and then induced with β-d-1-thiogalactopyranoside (1 mM IPTG) at 37 °C for 4 h. The cells were harvested by centrifugation (6000× *g* for 20 min at 4 °C) and resuspended in a lysis buffer (20 mM Tris HCl, 250 mM NaCl, and 0.1 mM EDTA, pH 8.0). Then, the suspension was incubated with 100 µg/mL lysozyme at room temperature for 30 min, mixed with 1 mM protease inhibitor phenylmethylsulfonyl fluoride (PMSF), and disrupted by sonication. The cell debris was removed by centrifugation (10000× *g* for 30 min at 4 °C), and the supernatant was filtered through a 0.2-µm filter. The proteins were purified on an NGC Discovery^TM^ 10 FPLC system (Bio-Rad, Hercules, CA, USA) with a HisTrap FF column (GE Healthcare, Solingen, Germany) pre-charged with Ni^2+^ ions. The filtered lysate was supplemented with imidazole and MgCl_2_ to a final concentration of 50 mM and 1 mM, respectively, and loaded on a column preequilibrated with a binding buffer (20 mM Tris HCl, 250 mM NaCl, and 50 mM imidazole, pH 8.0). The fractions were eluted using a linear gradient to a 100% elution buffer (20 mM Tris HCl, 250 mM NaCl, and 500 mM imidazole pH 8.0). The collected protein fractions were dialyzed against 20 mM Tris HCl pH 7.5.

The purity of the proteins was determined by 16% SDS-PAGE. The protein concentrations were measured using a spectrophotometer (Implen NanoPhotometer, IMPLEN, München, Germany) at 280 nm and calculated using a predicted extinction coefficient (1.46 (mg/mL)^-1^ cm^-1^).

### 2.4. Evaluation of the K. pneumoniae Antibiotic Susceptibility

*K. pneumoniae* strain Ts 141-14 susceptibility to antibiotics (ampicillin, cefotaxime, ceftazidime, meropenem, gentamicin, amikacin, ciprofloxacin, tetracycline, and chloramphenicol) was performed by the broth microdilution method using Mueller-Hinton broth, according to ISO recommendations (ISO. ISO 20776-1. Clinical laboratory testing and in vitro diagnostic test systems—Susceptibility testing of infectious agents and evaluation of performance of antimicrobial susceptibility testing devices—part 1. Geneva, Switzerland: International Organization for Standardization, 2006.). Results were interpreted according to The European Committee on Antimicrobial Susceptibility Testing. Breakpoint tables for interpretation of MICs and zone diameters. Version 10.0, 2020. (http://www.eucast.org). *Escherichia coli* strains ATCC 25922 and ATCC 35218 were used for quality control.

### 2.5. In Vitro Antibiofilm Activity

Recombinant endolysin LysECD7 fused to an 8-His tag at the C-terminus was freeze-dried for long term storage at +4 °C. LysECD7 was reconstituted in a sterile 20 mM Tris-HCl buffer (pH 7.5) immediately before use.

Evaluation of the antibiofilm activity of the LysECD7 was assessed during biofilm formation and on mature biofilms. In both experiments 100 µL of suspensions of *K. pneumoniae* Ts 141-14 in PBS buffer (pH 7.5) containing 1 × 10^9^ CFU/mL (McFarland standard 3.0) were mixed with 100 µL of the GRM 1 medium in 96-well sterile polystyrene cell culture plates (flat bottom with lid, Costar Corning, NY, USA).

Following preparations were investigated: LysECD7 in concentrations 1000 and 3000 µg/mL (62 and 186 µM correspondingly); amikacin in concentrations 50, 100 and 250 µg/mL (64, 128 and 320 µM respectively). Untreated cells, cells incubated in PBS or 20 mM Tris-HCl buffer (pH 7.5) were used as control groups.

To assess activity against biofilm formation, the investigated or control solutions (100 µL) were added immediately after preparation of the culture mix (200 µL) and incubated for 24 h at 37 °C without agitation.

To assess activity against mature biofilms the culture mix (200 µL) was pre-incubated for 24 h at 37 °C without agitation, rinsed three times with PBS (pH 7.5), added GRM 1 broth (100 µL) and investigated or control solutions (100 µL) and incubated for 19 h at 37 °C without agitation.

The planktonic cells were removed at the end of the cultivation in both experiments by triple rinsing with sterile PBS buffer. Washed biofilms were stained with 0.1% aqueous solution of crystal violet for 15 min at room temperature without agitation followed by triple rinsing with sterile PBS. Remained crystal violet was eluted with 200 µL 95% ethanol and obtained solution was transferred to clean flat-bottomed plates for following measurement of the optical density at a wavelength of 590 nm using xMark™ Microplate Absorbance Spectrophotometer (Bio-Rad, Hercules, CA, USA). The results were interpreted according to the optical density of the colored solvent [21]. All experiments were done in triplicates.

The interpretation of the level of biofilm formation was done accordingly to Stepanovic et al., 2007 [21]. Briefly, weak biofilm was defined at ODc < OD_Kp_ ≤ 2×ODc, moderate biofilm at 2×ODc < OD_Kp_ ≤ 4×ODc, and strong biofilm at 4 × ODc < OD_Kp_, where ODc is the cut-off value calculated as three standard deviations (SD) above the mean OD of the negative control, OD_Kp_ = the optical density of *K. pneumoniae* Ts 141-14 well stained with crystal violet.

### 2.6. In Vivo Model

#### 2.6.1. Diffusion Chambers

For safe and representative in vivo modeling of the bacterial biofilm we used diffusion chambers implanted in outbred rats. The chambers were made of acrylic frame (internal ∅ 10 mm, external ∅ 20 mm, thickness 5 mm) with an inlet port (∅ 0.6 mm) sealed with a nitrocellulose membrane (pore size ∅ 0.15 µm) MFAS–B–3 (Vladipor, Vladimir, Russia) (Figure S1). Tightness of the chambers did not allow bacterial cells to leave the chamber after implantation into the animal’s body. Diffusion chambers were successfully used for assessment of antibacterial substances in vivo [22,23]. However, capabilities of this perspective model were not tested in biofilm related studies.

Sterile diffusion chambers were filled with 200 µL of freshly prepared PBS-suspension of the *K. pneumoniae* Ts 141-14 (OD_595_ = 0.2; 4.56 × 10^8^ ± 6.84 × 10^7^ CFU/mL) by a syringe through the inlet port followed by sealing with a piece of the membrane within one hour before an implantation.

#### 2.6.2. Experimental Animals

In total thirty outbred white rats (female, 4 months old, weight range 250–270 g) were used for in vivo studies. All animals received care in accordance with the Guidelines for accommodation and care of animals (GOST 33215-2014 “Environment, housing and management” and GOST 33216-2014 “Species-specific provisions for laboratory rodents and rabbits”, Moscow, Russia) which corresponds to the ETS No. 123 “European Convention for the Protection of Vertebrate Animals used for Experimental and Other Scientific Purposes”. The in vivo studies were approved and permitted by the Ethics Committee of the Astrakhan State Medical University (Record No. 5, 06.06.2019, Astrakhan, Russia) followed by carrying out at the Astrakhan State Medical University with commitment to the 3Rs principles. The animals were kept at the laboratory under veterinary supervision in individual cages at normal conditions for the rodents (room temperature of +22 °C ± 2 °C; humidity of 50% ± 5%) and a standard diet as well as water ad libitum were provided during the entire study.

#### 2.6.3. Implantation and Explantation of Diffusion Chambers

Prior to all surgical procedures a general anesthesia was induced by inhalation of diethyl ether and maintained during surgery. Followed by a fixation on the operating table, the median laparotomy was performed and the diffusion chambers were introduced into the abdominal cavity. A continuous Reverdin suture was applied to the external aponeurosis lamellae of the rectus abdominis muscle and a vertical mattress stitch was used on the skin. Surgical silk was used as a suture material with a cutting needle C-20. The anesthesia was stopped after the surgery and the animals were released from the fixing dressings, placed in clean cages and carried out in accordance with experimental protocols. The animals were euthanized following explantation of the chambers. All surgical procedures were performed under aseptic conditions.

#### 2.6.4. Estimation of In Vivo Biofilm Formation

Preliminary study in three rats was conducted in order to define a time point of initiation of an antibiofilm therapy. Following parameters were assessed before, 2, 4 and 6 days after the implantation of diffusion chambers: general clinical condition, postoperative wound condition, visual inflammation of surrounding tissues, sterility of the surrounding tissues, sterility of the implant external surface, visual characteristics of the frame inner surface, visual characteristics of the membrane inner surface, and presence of the specific *K. pneumoniae* growth from the cavity of the chamber.

#### 2.6.5. In Vivo Assessment of the LysECD7 on Preformed Biofilms

**Figure 1:**
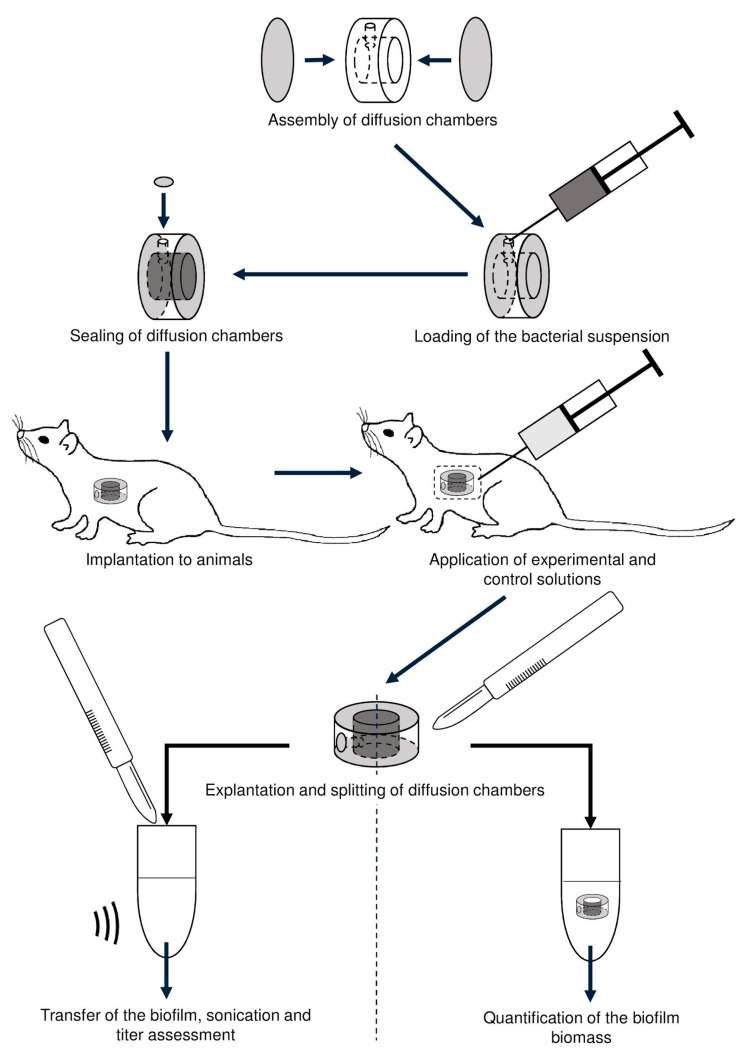
Experimental pipeline of the in vivo study.

The presented scheme (Figure 1) outlines the procedures and experiments performed to assess antibiofilm action of the LysECD7 (100 µg/mL) in comparison with amikacin (5 mg/mL) and 20 mM Tris-HCl buffer (pH 7.5) as a control.

Either LysECD7, amikacin, or 20 mM Tris-HCl buffer (pH 7.5) were injected intraperitoneally in a volume of 500 µL daily during seven days, beginning from the 4th day after chamber implantation, avoiding perforation of the membranes of diffusion chambers in the time point defined in the preliminary study. Three animals from each group were discontinued from the therapy on the 2nd, 5th, and 8th day followed by visual characteristics and sterility testing of tissues surrounding the implant, explantation of the chambers and sterility assessment of their external surface, removal of the membrane, and triple rinsing of the chamber with PBS. The explanted chambers were disclosed and analyzed for BF staining (BF biomass measurement) and culturable cells count.

#### 2.6.6. Biofilm Biomass Measurement

Explanted and washed diffusion chambers were placed in 3 mL of 0.1% aqueous solution of crystal violet for 10 min at room temperature without stirring followed by triple rinsing with sterile PBS. Remained crystal violet was eluted with 3 mL of 1:1 mixture of ethanol:isopropanol and obtained solution in a volume of 200 µL was transferred to clean flat-bottomed plates for following measurement of the optical density at a wavelength of 595 nm using iMark™ Microplate Absorbance Reader (Bio-Rad, Hercules, CA, USA).

#### 2.6.7. Assessment of Amount of Culturable Bacteria

Visible biofilm from ½ of the frame inner surface (ca. 78.5 mm^2^) was scraped out with a sterile surgical scalpel and transferred to 1 mL of PBS followed by sonication (20 min, room temperature, 22 kHz, 75-110 W) in order to disaggregate the biofilm [24]. The bacterial suspensions were serially diluted followed by plating out on the CHROMagar Orientation (CHROMagar, Paris, France) dishes and overnight incubation at +37 °C for CFU assessment.

#### 2.6.8. Sterility Assessment

Sterility tests were done by imprint smears from surfaces of the chambers and surrounding tissues. The chambers were removed with sterile forceps followed by application to the surface of the CHROMagar Orientation (CHROMagar, Paris, France). Another sterile forceps and scissors were used to cut off a piece of the surrounding peritoneum for the sterility test.

Swabs from the internal content of the chambers were performed with sterile inoculation loops and plated out on the CHROMagar Orientation (CHROMagar, Paris, France) in order to assess the *K. pneumoniae* culture purity.

### 2.7. Statistical Analysis

Data were analyzed using GraphPad Prism 8. The data are summarized using mean values and SDs of three independent experiments. Nonparametric statistical tests were used to calculate significance in all the experiments. *p* < 0.05 was considered significant.

## 3. Results and Discussion

### 3.1. LysECD7 Is Active against K. pneumoniae Biofilm In Vitro

**Figure 2:**
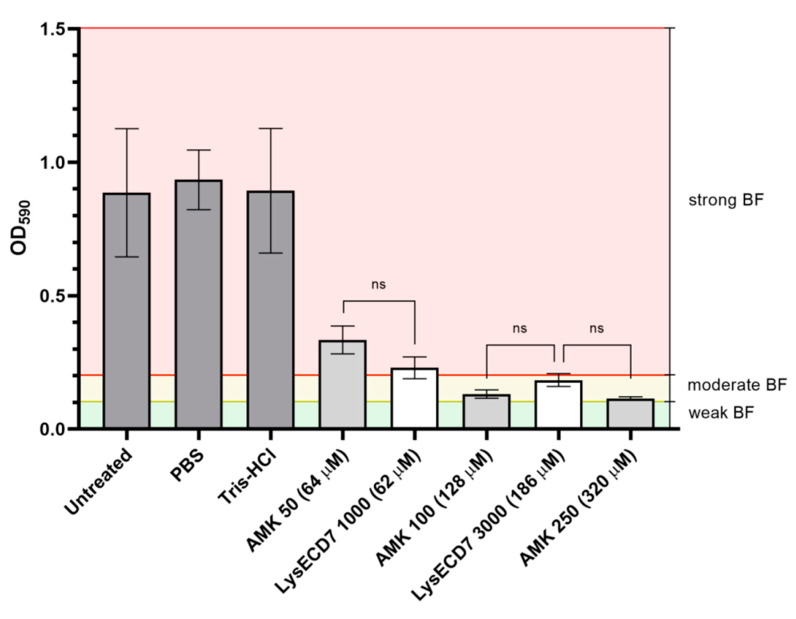
Antibacterial activity of the LysECD7 and amikacin (AMK) against forming biofilm of *K. pneumoniae* Ts 141-14 measured after 24 h of culture incubation; Untreated—untreated BF; PBS—BF treated with Phosphate-Buffered Saline; Tris-HCl—BF treated with 20 mM Tris-HCl buffer; AMK 50 (64 µM)—forming BF treated with 50 µg/mL AMK; LysECD7 1000 (62 µM)—forming BF treated with 1000 µg/mL LysECD7; AMK 100 (128 µM)—forming BF treated with 100 µg/mL AMK; LysECD7 3000 (186 µM)—forming BF treated with 3000 µg/mL LysECD7; AMK 250 (320 µM)—forming BF treated with 250 µg/mL AMK. Weak, moderate and strong BF were estimated according to Stepanovic et al., 2007 [21]. Data are shown as mean ± standard deviation; “ns”—no significant differences were observed (one-way ANOVA with Dunnett’s multiple comparisons test). Differences between treatments and controls were significant (*p* < 0.0001).

Endolysins actively degrade cell wall peptidoglycans, resulting in quick lysis of bacterial cells and do not require active bacterial metabolism to exert their bactericidal effect. That means that they can target not only metabolically active planktonic cells but also persistent bacteria causing chronic infections and their BF [8]. *K. pneumoniae* is a big clinical problem in the field of organ transplantation and implanted medical devices. Its virulence and antimicrobial resistance significantly modulated by biofilm formation properties [25]. In the frame of the present study, *K. pneumoniae* strain Ts 141-14 has been chosen as a model because of its biofilm production capacities and virulent phenotype in our rat animal model. The in vitro activity of LysECD7 against forming and mature biofilms was investigated on the *K. pneumoniae* Ts 141-14 strain which appeared to be a strong biofilm producer as measured in microtiter plates test according to Stepanovic et al., 2007 [21]. We also compared the effect of endolysins action with amikacin treatment, which was the only antibiotic active against the *Klebsiella* test strain (Table S1). The antibacterial effect of LysECD7 against forming and mature biofilm of *K. pneumoniae* strain Ts 141-14 was evaluated by the crystal violet staining assay. It was shown that LysECD7 in concentrations 1000 and 3000 µg/mL (62 and 186 µM) prevented biofilm formation of 74% and 79%, respectively, compared to the control groups. At the same time, amikacin, in concentrations 50, 100, and 250 µg/mL (64, 128 and 320 µM) also reduced the biofilm density (Figure 2). Thus, in a close concentration range the same effect can be seen both for LysECD7 and amikacin.

**Figure 3:**
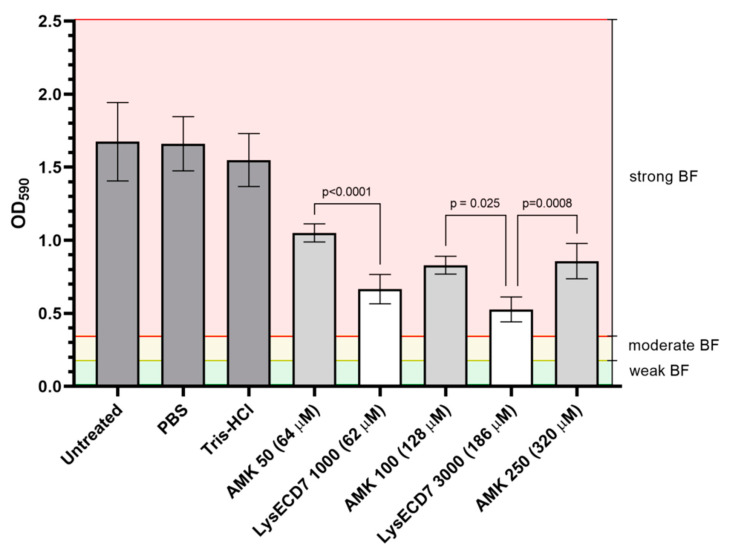
Antibacterial activity of the LysECD7 and amikacin (AMK) against mature BF measured after 43 h of culture incubation of *K. pneumoniae* strain Ts 141-14; Untreated—untreated BF; PBS—BF treated with Phosphate-Buffered Saline; Tris-HCl—BF treated with 20 mM Tris-HCl buffer; AMK 50 (64 µM)—mature BF treated with 50 µg/mL AMK; LysECD7 1000 (62 µM)—mature BF treated with 1000 µg/mL LysECD7; AMK 100 (128 µM)—mature BF treated with 100 µg/mL AMK; LysECD7 3000 (186 µM)—mature BF treated with 3000 µg/mL LysECD7; AMK 250 (320 µM)—mature BF treated with 250 µg/mL AMK. Weak, moderate and strong BF were estimated according to Stepanovic et al., 2007 [21]. Data are shown as mean ± standard deviation. Significant differences with the AMK groups are shown as *p*-values (one-way ANOVA with Dunnett’s multiple comparisons test). Differences between treatments and controls were significant (*p* < 0.0001).

The disruption activity of LysECD7 against mature BF in concentrations 1000 and 3000 µg/mL (62 and 186 µM) was higher than disruption activity of amikacin 50–250 µg/mL (64–320 µM): BF-density was decreased on 60% and 68% compared to 37%, 50%, and 49%, in comparison with the control groups respectively (Figure 3).

Thus, LysECD7 is more effective against forming biofilms than mature biofilms, but more efficient than amikacin against the latter. Amikacin therapeutic concentrations (11 to 15 mg/kg/day) normally used for Gram-negative bacteremia treatment is associated with different adverse events, however higher doses may be required to manage patients with sepsis [26]. In this case, the activity of endolysin is comparable to the action of the antibiotic and can be useful for therapeutic use alone or in combination with antibiotics to reduce the AB doses.

The concentrations of 62 and 186 µM are fairly high doses for therapeutic use. However, we have shown previously bactericidal activity in vitro of the LysECD7 against planktonic cells of Gram-negative bacteria in concentrations varying from 0.5 to 100 µg/mL (0.031–6.2 µM) [19,20]. Among others it was shown that 100 μg/mL (6.2 µM) was enough to eradicate growing *K. pneumoniae* strain Ts 104-14 and reduced up to 7.61 × 10^3^ CFU/mL [20]. Furthermore, the determined LysECD7 MIC value was > 1 mg/mL. For other endolysins, effective concentrations up to 500 µg/mL [11,13,27] could be found in the literature while the values significantly depended on the strains used.

### 3.2. In Vivo Biofilm Formation

To define a time point of initiation of an antibiofilm therapy, a preliminary study in three rats was conducted. Dynamics of the biofilm formation in vivo were investigated and summarized in Table 1.

It was shown that the optimal time point for the beginning of therapy was the fourth day after implantation of diffusion chambers (Table 1): before this time point, no visual signs BF formation were observed. At the later date, the general clinical condition of the animal worsens. Primary intention is a healing of uninfected postoperative wounds with approximated edges by sutures [28], which was observed beginning from the second day after implantation.

### 3.3. In Vivo Efficacy of the LysECD7

#### 3.3.1. Intraoperative Clinical Picture

**Figure 4:**
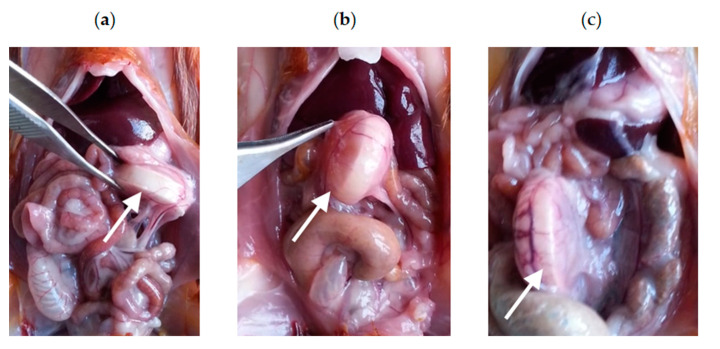
Intraoperative view of the diffusion chambers in the abdominal cavity on the 8th day of administration of: (**a**) LysECD7 (100 µg/mL), (**b**) amikacin (5 mg/mL), (**c**) vehicle control.

In vivo anti-BF activity of LysECD7 was assessed using 50 µg of the endolysin per animal daily for seven days, beginning from the fourth day after chamber implantation. Rats with administration of 20 mM Tris-HCl pH 7.5 was used as a vehicle control group. The AMK was used for comparison in the concentration recommended by the manufacturer’s instruction (2.5 mg per animal). To observe the dynamics of endolysin treatment a post-mortem examination (autopsy) of three animals at each point was performed: on the second, fifth and eighth day after the beginning of treatment (Figures S2 and S3, Figure 4). On the second day of the LysECD7 administration slight inflammatory infiltration of tissues surrounding the chamber was observed (Figure S2a). Administration of the amikacin at this time point protected the animals from any visual signs of periimplantitis (Figure S2b). The animals in the vehicle control group showed inflammatory infiltration of tissues surrounding the chamber and exudate of the peri implant zone (Figure S2c).

On the fifth day of the LysECD7 administration no visual differences were observed in comparison with the previous time point (Figure S3a). Minor inflammatory infiltration appeared on the fifth day of the AMK application (Figure S3b). Intraoperative picture remained constant (inflammatory infiltration of tissues surrounding the chamber and exudate of the peri implant zone) for the animals obtaining Tris-HCl buffer only (Figure S3c).

The most significant differences were observed on the eighth day, when either slight or no inflammatory infiltration was detected in rats treated with LysECD7 (Figure 4a), while inflammatory infiltration of tissues surrounding the chamber and exudate of the peri implant zone was shown in both AMK and vehicle control groups at this time point (Figure 4b,c).

#### 3.3.2. Biofilms Development Dynamics

**Figure 5:**
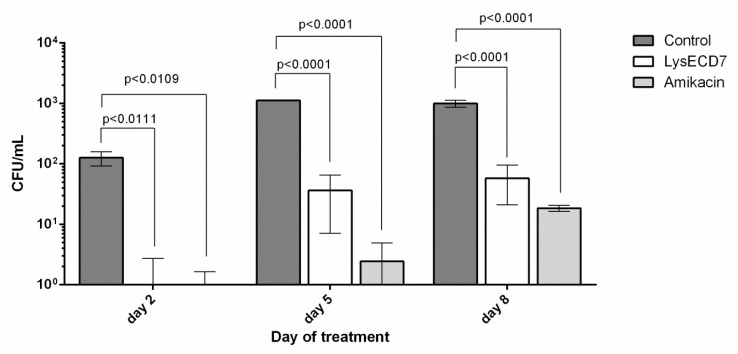
Count of culturable bacteria in the formed biofilms in diffusion chambers in vivo during performed therapy. Data are shown as mean ± standard deviation. Significant differences with the vehicle control group are shown as *p* values, otherwise no statistical difference was found (two-way ANOVA with Dunnett’s multiple comparisons test). On the 5^th^ day, a significant difference between endolysin and antibiotic groups was also detected (*p* = 0.0001).

Number of culturable bacteria in the biofilms in vivo showed statistically significant differences on the second day of therapy between vehicle control, LysECD7 and AMK groups of animals (Figure 5). At the fifth day of treatment the bacteria count reduced from 1.1 × 10^3^ ± 7.2 CFU/mL in the control group to 36.3 ± 29.2 CFU/mL and 2.4 ± 2.5 CFU/mL in the endolysin and antibiotic groups, respectively. On the eighth day of treatment, the colony count decreased from 1.0 × 10^3^ ± 128.1 CFU/mL to 58.0 ± 37.0 CFU/mL and 18.5 ± 2.1 CFU/mL correspondingly. Up to 10^3^ in the number of viable bacteria in the formed biofilms in diffusion chambers was disrupted by 50 μg of LysECD7 injected intraperitoneally. Thus, CFU counts were significantly lower in animals treated with LysECD7 or AMK in comparison with the control group during the entire experiment; however, no significant differences were observed between the first two groups.

**Figure 6:**
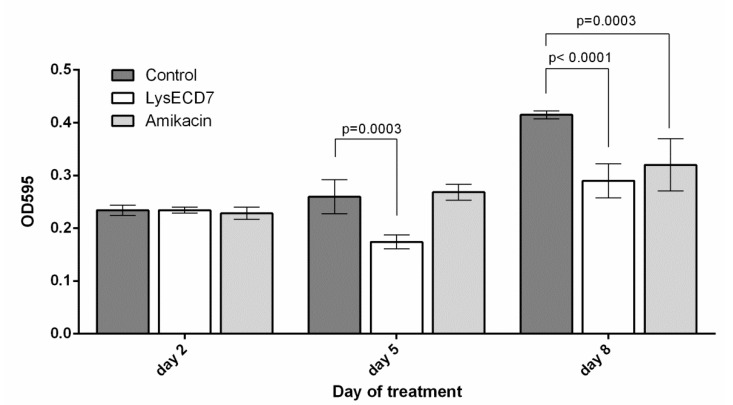
Dynamics of the biomass of biofilms in diffusion chambers in vivo during performed therapy. Data are shown as mean ± standard deviation. Significant differences with the vehicle control group are shown as *p* values, otherwise no statistical difference was found (two-way ANOVA with Dunnett’s multiple comparisons test).

The stained biofilm optical density values did not differ on the second day of therapy between all groups (Figure 6). On the fifth day of therapy, they were significantly lower for the LysECD7 group in comparison with the control group while AMK-treated animals did not differ from the control. The OD_595_ differences on the eighth day of experiment showed the same significance as the CFU count at this time point.

Surfaces of the diffusion chambers and surrounding tissues remained sterile in vivo during the entire experiment in almost all animals. One rat in the amikacin group was excluded from the analysis due to failure of a junction between the acrylic frame and the membrane at the last time point (Tables S2 and S3).

Endolysin’s ability to decrease BF formation and to eradicate mature BF is of special interest not only in the sphere of transplantology but also in BF-related infections such as urinary tract, orthopedic or oral infections. Previously, endolysins targeting Gram-positive bacteria were tested for their ability to eradicate staphylococcal biofilm infections, including MRSA strains in vitro. It was possible to detect a decrease in the number of BF bacteria by more than 10^5^ CFU/mL in vitro proposing eradication of all biofilm biomass [29,30,31]. Among enzymes targeting Gram-negative bacteria, anti-BF activity was shown in vitro against *Pseudomonas aeruginosa:* LysPA26 was capable of disrupting about 10^1^ to 10^2^ of viable counts of biofilm cells [32] and *Acinetobacter baumannii* both in vitro and in vivo reducing up to 10^2^ of bacterial viability in catheter-based infection model [13]. LysECD7 showed similar results in experiments eradicating 10^2^–10^3^ of bacterial load of implanted diffusion chambers.

Gutiérrez et al. evaluated in vitro activity against bacterial biofilms, but the target was Gram-positive bacteria (*Staphylococcus spp.*) [33]. It should be mentioned, that for Gram-negative targeting endolysins the concentrations used are normally greater: 500 µg/mL [32] and 1 mg/mL [13]. Since, for LysECD7, MIC value was more than 1 mg/mL we used this concentration as a start point for experiments. However, as can be seen from the in vivo model, concentrations can be significantly reduced since even 100 µg/mL (6.2 µM) gave a visible effect in bacterial load reduction and biomass of biofilms.

Thus, LysECD7 possesses an ability to act against emerging and formed biofilms both in vitro and in vivo, comparable with the activity of AMK but with less risks of inflammatory infiltration of abdominal cavity tissues and exudate accumulation in the peri implant zone. This indicates that LysECD7 can be considered as a promising agent with strong antibiofilm activity suitable for local or systemic applications.

## Figures and Tables

**Table 1:** Comparative characteristics of the *K. pneumoniae* Ts 141-14 biofilms formation in vivo.

Indicator	Before Implantation	Two Days After Implantation	Four Days After Implantation	Six Days After Implantation
General clinical condition	Not changed	Not changed	Not changed	The animal is inactive, with a lack of appetite
Postoperative wound condition	–	Primary intention healing	Primary intention healing	Primary healing, slight inflammation of the postoperative wound
Visual inflammation of surrounding tissues	–	Slight inflammatory infiltration of tissue surrounding the chamber	Absent	Inflammatory infiltration of tissues surrounding the chamber, peri implant zone exudate
Sterility of the surrounding tissues	–	Sterile	Sterile	Sterile
Sterility of the implant external surface	Sterile	Sterile	Sterile	Sterile
Visual characteristics of the frame inner surface	No visible BF	No visible BF	The presence of mucous BF	The presence of a dense, non-stretching BF
Visual characteristics of the membrane inner surface	No visible biofilm	No visible biofilm	No visible biofilm	The presence of a dense, non-stretching biofilm

The sign “-“ corresponds to not applicable indicatiors.

## Supplementary Materials

The following are available online at https://www.mdpi.com/1999-4915/12/5/545/s1. Table S1: *K. pneumoniae* strain Ts 141-14 MIC distribution for antibiotics; Table S2: Count of culturable bacteria in the formed biofilms in diffusion chambers in vivo during the therapy; Table S3: Dynamics of the biofilms density in diffusion chambers in vivo during the therapy; Figure S1: Schematic structure of the diffusion camera; Figure S2: Intraoperative view of the diffusion chambers in abdominal cavity on the 2nd day of treatment; Figure S3: Intraoperative view of the diffusion chambers in the abdominal cavity on the 5th day of treatment; Figure S4: Antibacterial activity of amikacin (AMK).
