# Supplementary material for: Antibiofilm Activity of a Broad-Range Recombinant Endolysin LysECD7: In Vitro and In Vivo Study
Source: Viruses. 2020 May 15;12(5):545. doi: 10.3390/v12050545 (PMC7291189; doi:10.3390/v12050545)
Supplement: Supplementary file 1 [file viruses-12-00545-s001.pdf]

Supplementary materials

Antibiofilm activity of a broad-range recombinant endolysin LysECD7: *in vitro* and *in vivo* study

Mikhail V. Fursov [1,§], Radmila O. Abdrakhmanova [2,§], Nataliia P. Antonova [3,4, §], Daria V. Vasina [3,5, §], Anastasia D. Kolchanova [1], Olga A. Bashkina [2], Oleg V. Rubalsky [2], Marina A. Samotrueva [2], Vasiliy D. Potapov [1], Valentine V. Makarov [6], Sergey M. Yudin [6], Alexander L. Gintsburg [3,7], Artem P. Tkachuk [3], Vladimir A. Gushchin [3,4,&,*], Evgenii O. Rubalskii [8,9,10,&,*]

Table S1. Minimal inhibitory concentration (MIC) of different antibiotics against *Klebsiella pneumoniae* strain TS 141-14

Antibacterials	Group	MIC, mg/L	Interpretation
	Beta-lactam antibiotics		
Ampicillin	Penicillins	>256	R
Cefotaxime	Cephalosporins III Generation	>256	R
Ceftazidime	Cephalosporins III Generation	>256	R
Meropenem	Carbapenems	>256	R
	Aminoglycosides		
Gentamicin	Aminoglycosides	128	R
Amikacin	Aminoglycosides	4	S
	Others		
Ciprofloxacin	Fluoroquinolones	>256	R
Tetracycline	Tetracyclines	>256	R
Chloramphenicol		>256	R

Note: R – resistant; S – sensitive

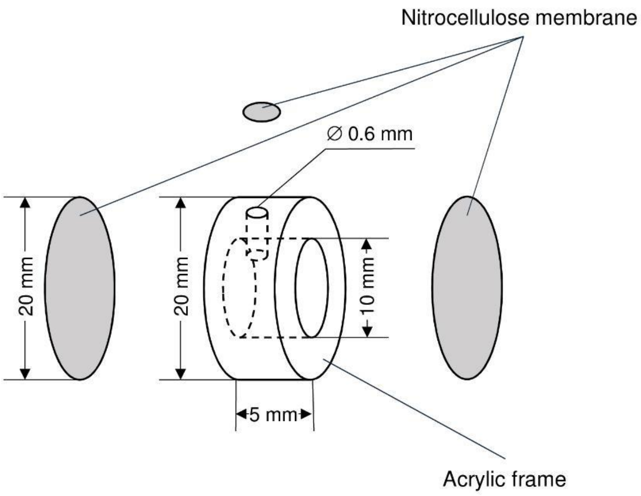

Figure S1. Schematic structure of the diffusion camera

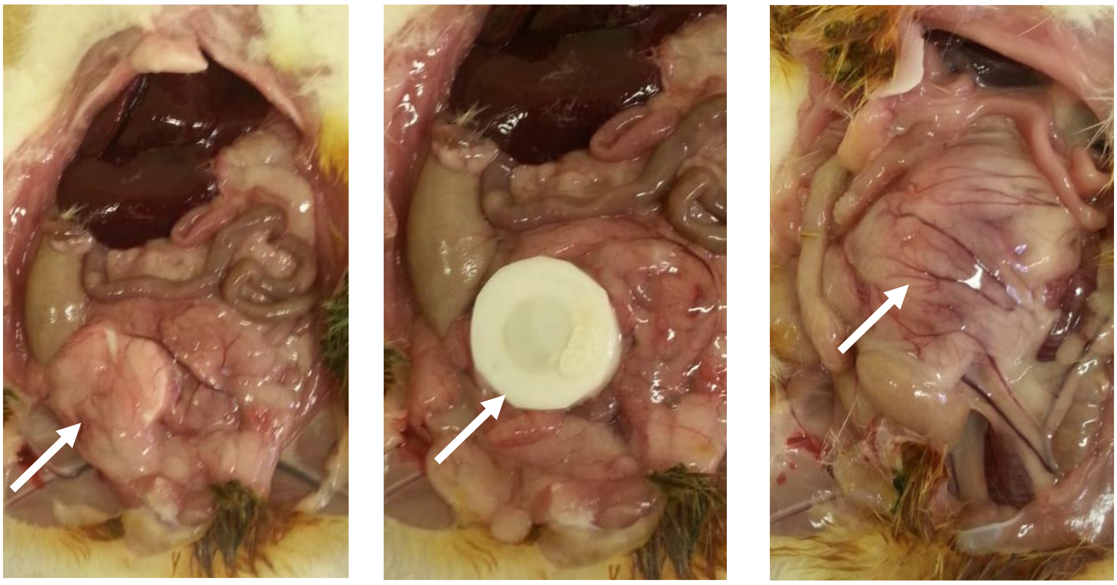

(**a**) (**b**) (**c**)

Figure S2. Intraoperative view of the diffusion chambers in the abdominal cavity on the 2nd day of treatment with: (**a**) LysECD7. (**b**) amikacin. (**c**) Tris-HCl buffer.

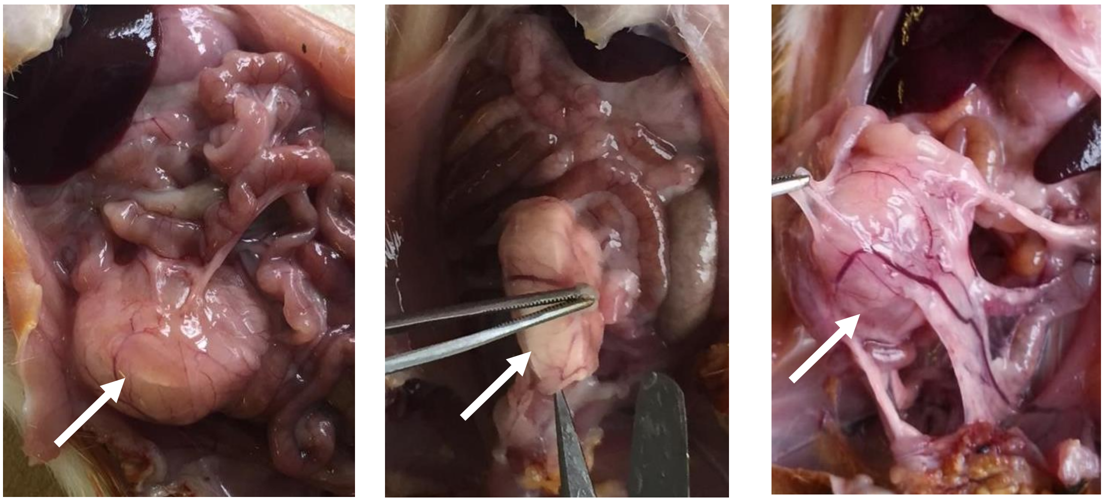

(**a**) (**b**) (**c**)

Figure S3. Intraoperative view of the diffusion chambers in the abdominal cavity on the 5th day of treatment with: (**a**) LysECD7. (**b**) amikacin. (**c**) Tris-HCl buffer.

Table S2. Count of culturable bacteria in the formed biofilms in diffusion chambers in vivo during the therapy

Day of application	CFU/mL								
	LysECD7			Amikacin			Control		
2	0	3	0	0	0	1.80	$1.63 \cdot 10^{2}$	$1.17 \cdot 10^{2}$	$9.80 \cdot 10^{1}$
5	$7.00 \cdot 10^{1}$	$1.80 \cdot 10^{1}$	$2.10 \cdot 10^{1}$	0	5	2.3	$1.11 \cdot 10^{3}$	$1.12 \cdot 10^{3}$	$1.12 \cdot 10^{3}$
8	$1.00 \cdot 10^{2}$	$3.00 \cdot 10^{1}$	$4.40 \cdot 10^{1}$	$2.00 \cdot 10^{1}$	$1.70 \cdot 10^{1}$	$7.20 \cdot 10^{2}$*	$8.63 \cdot 10^{2}$	$1.12 \cdot 10^{3}$	$1.01 \cdot 10^{3}$

* Failure of diffusion chamber integrity

Table S3. Dynamics of the biofilms density in diffusion chambers *in vivo* during the therapy

Day of application	OD_{595}								
	LysECD7			Amikacin			Control		
2	0.234	0.240	0.229	0.234	0.215	0.236	0.232	0.226	0.245
5	0.179	0.184	0.159	0.282	0.252	0.271	0.223	0.274	0.282
8	0.275	0.268	0.327	0.355	0.285	0.346*	0.414	0.408	0.423

* Failure of diffusion chamber integrity

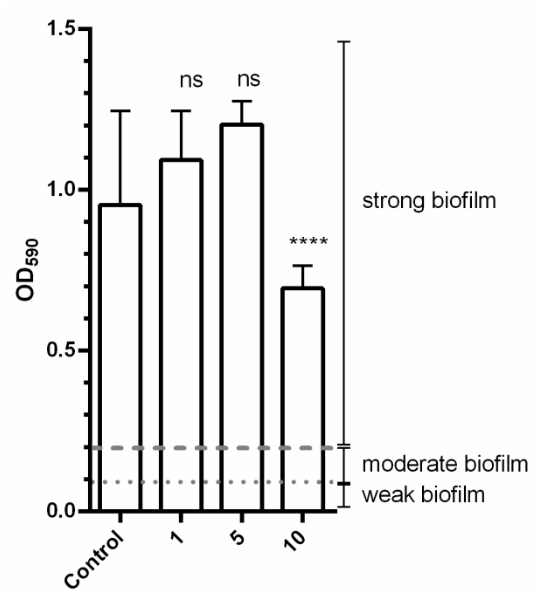

(**a**)

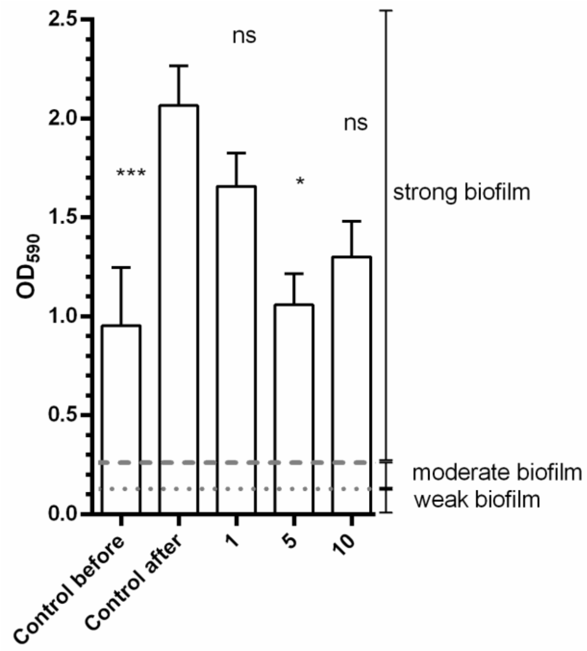

(**b**)

Figure S4. Antibacterial activity of amikacin (AMK) against: (**a**) forming biofilm of *K. pneumoniae* Ts 141-14 measured after 24 h of culture incubation: Control - untreated BF after 24 h of culture incubation; 1 - forming BF treated with 1 μg/mL AMK; 5 - forming BF treated with 5 μg/mL AMK; 10 - forming BF treated with 10 μg/mL AMK; (**b**) mature BF measured after 43 h of culture incubation of *K. pneumoniae* strain Ts 141-14. Control before - untreated BF after 24 h of culture incubation; Control after - untreated BF after 43 h of culture incubation; 1 - mature BF treated with 1 μg/mL AMK; 5 - mature BF treated with 5 μg/mL AMK; 10 - mature BF treated with 10 μg/mL AMK.
